# Genome-wide characterization of NtHD-ZIP IV: different roles in abiotic stress response and glandular Trichome induction

**DOI:** 10.1186/s12870-019-2023-4

**Published:** 2019-10-24

**Authors:** Hongying Zhang, Xudong Ma, Wenjiao Li, Dexin Niu, Zhaojun Wang, Xiaoxiao Yan, Xinling Yang, Yongfeng Yang, Hong Cui

**Affiliations:** 1 College of Tobacco Science, Henan Agricultural University, Zhengzhou, 450002 China; 2 Technology Center, China Tobacco Henan Industrial Co., Ltd, Zhengzhou, 450000 China

**Keywords:** *Nicotiana tabacum*, Expression pattern, MeJA, ABA, GA, SA

## Abstract

**Background:**

The plant-specific homeodomain-leucine zipper class IV (HD-ZIP IV) gene family has been involved in the regulation of epidermal development.

**Results:**

Fifteen genes coding for HD-ZIP IV proteins were identified (*NtHD-ZIP-IV-1* to *NtHD-ZIP-IV-15*) based on the genome of *N. tabacum*. Four major domains (HD, ZIP, SAD and START) were present in these proteins. Tissue expression pattern analysis indicated that *NtHD-ZIP-IV-1, − 2*, *− 3*, *− 10*, and *− 12* may be associated with trichome development; *NtHD-ZIP-IV-8* was expressed only in cotyledons; *NtHD-ZIP-IV-9* only in the leaf and stem epidermis; *NtHD-ZIP-IV-11* only in leaves; and *NtHD-ZIP-IV-15* only in the root and stem epidermis. We found that jasmonates may induce the generation of glandular trichomes, and that *NtHD-ZIP-IV-1, − 2, − 5,* and *− 7* were response to MeJA treatment. Dynamic expression under abiotic stress and after application of phytohormones indicated that most *NtHD-ZIP IV* genes were induced by heat, cold, salt and drought. Furthermore, most of these genes were induced by gibberellic acid, 6-benzylaminopurine, and salicylic acid, but were inhibited by abscisic acid. *NtHD-ZIP IV* genes were sensitive to heat, but insensitive to osmotic stress.

**Conclusion:**

*NtHD-ZIP IV* genes are implicated in a complex regulatory gene network controlling epidermal development and abiotic stress responses. The present study provides evidence to elucidate the gene functions of *NtHD-ZIP IV*s during epidermal development and stress response.

## Background

Plants have developed a complex regulatory network to adapt to extreme environmental stresses, in which jasmonic acid (JA), salicylic acid (SA) and abscisic acid (ABA) act as pivotal defense signal molecules [1–3]. Plant trichomes are involved in defense responses towards insect predation, UV damage, toxin sequestration, and excess transpiration. Trichomes are grouped into two types, glandular and non-glandular. Glandular trichomes can synthesize and secrete large numbers of specialized metabolites, including terpenes, phenylpropanoids, sucrose esters, and flavonoids [4, 5]. These natural plant compounds not only protect plants against insect pests, but also contribute to the production of industrial chemicals for use in flavors, aromas, and pharmaceuticals [6–8]. In Arabidopsis, it was reported that exogenous application of JA and GA induced the occurrence of non-glandular trichomes [9]. In tomato, exogenous application of JA resulted in a dramatic increase in glandular trichome density [10].

The plant-specific homeodomain-leucine zipper (HD-Zip) gene family plays a crucial role in abiotic stress response and plant development [11–13]. These proteins can be further grouped into 4 subfamilies according to their structural features, conserved domains, and physiological functions [[14–17]. The Class IV HD-Zip (hereafter “HD-Zip IV”) gene family is associated with lipid transport, epidermal development, cuticle biosynthesis, and anthocyanin deposition [18–20]. *HD-ZIP IVs* are also implicated in mediating plant defense to osmotic stress [21, 22]. In *Arabidopsis*, the HD-ZIP IV family comprises 16 genes; the first identified *HD-ZIP IV* gene (*GL2*) was implicated in root hair differentiation and trichome development [23, 24]. Two *AtHD-ZIP IVs, ML1* and *PDF2*, have been involved in regulating epidermis and embryo development and determining floral organ identity [25, 26]. One *AtHD-ZIP IV* gene, *AtANL2*, controls epidermal cell proliferation, root development, and anthocyanin accumulation [27]. Two closely-related and functionally-redundant *AtHD-ZIP IV*s, *HDG11* and *HDG12*, regulate branching of the trichome [19]. *HD-ZIP IV*s has been characterized in various groups other than *Arabidopsis*, namely maize, rice, soybean, and cucumber [18, 19, 28–30]. It was found that *HD-ZIP IVs* are primarily expressed in the epidermal tissue. Moreover, *Arabidopsis*, maize, rice, soybean, and cucumber possess only non-glandular trichomes. The recently published expression profile of *HD ZIP IVs* in tomatoes suggests that each member may fulfill distinct functions in plant development [31]. Up to now, the specific roles of *HD-ZIP IV*s in the induction of glandular trichomes has remained enigmatic.

The common tobacco (*Nicotiana tabacum*), a broadleaf crop with large yields and planting areas, has glandular trichomes on the surface of its leaves. These trichomes produce various terpenoids, alkaloids and defensive proteins, together representing up to 30% dry weight of the leaf [32–34]. Diterpenoids, including labdanoids and cembranoids, are more abundant in *Pinus* and *Nicotiana* than in other genera [35, 36]**.** In addition, cembranoids have neuroprotective, anti-microbial, and anti-tumor properties, and can help in the treatment of human immunodeficiency virus [37–40]. However, knowledge concerning the occurrence of glandular trichomes is fragmentary.

*N. tabacum* is an excellent model to clarify the gene functions of *HD-ZIP IVs* in dicotyledons. To elucidate the potential functions of *NtHD-ZIP IVs* in abiotic stress response and plant development, *N. tabacum HD-ZIP IV* genes were identified by the computational analysis of *N. tabacum* genome resource. We analyze gene structure, synteny, phylogeny, tissue expression pattern, and the expression profile under various exogenous hormones and abiotic stresses. In particular, we compare the transcript level of *HD-ZIP IVs* in the sub-epidermal and epidermal layers. Our study lays the foundation for characterization of *HD-ZIP IVs* in epidermis-related functions.

## Results

### Identification and analysis of *HD-ZIP IV* genes in *N. tabacum*

**Fig. 1:**
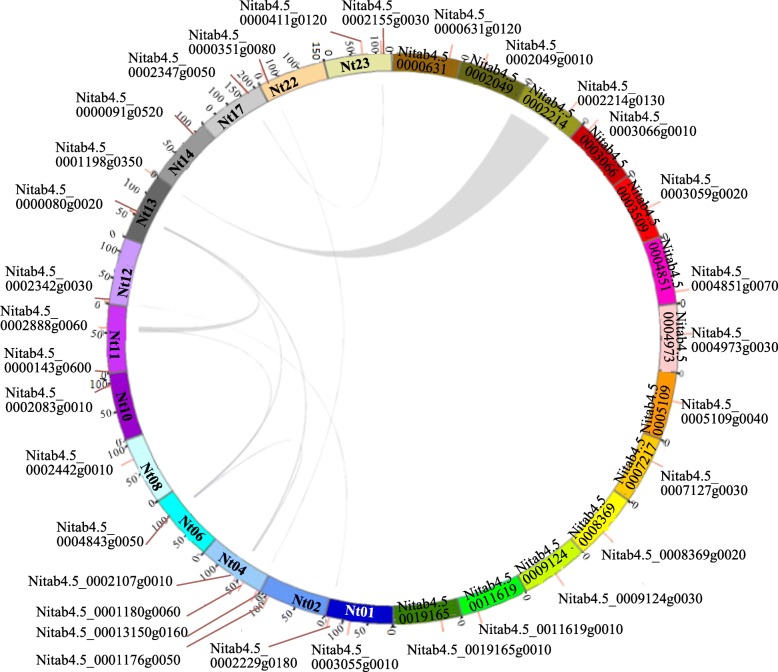
Chromosome distributions and synteny relationships of *HD-ZIP IVs* in *N. tabacum*. Gray lines show segmental duplications

Based on the latest genome data of tobacco, 32 *HD-ZIP IV* genes were identified in *N. tabacum* genomes. These HD-ZIP IV proteins had conserved domains namely HD, LZ, SAD and START. The positions of the *HD-ZIP IV* genes showed a scattered distribution pattern in the tobacco chromosome (Table 1). Chromosome 4 had three *HD-ZIP IV* gene copies, chromosomes 1, 11, 13, and 23 contained two copies, and chromosomes 2, 6, 8, 10, 12, 14, 17, and 22 individually had one copy. Moreover, nine pairs of *HD-ZIP IV*s were duplicated in tobacco genome (Fig. 1). The molecular weight of HD-ZIP IV proteins ranged from 49.66 to 91.77 kDa, the predicted full-length amino acid sequences ranged from 359 to 828, and the number of exons ranged from 4 to 11.

**Table 1:** HD ZIP IV gene family in *N. tabacum*

Gene locus	Location	Gene length (bp)	Exon Number	CDS (bp)	Length AA	Mw (KDa)	pI
Nitab4.5_0003055g0010	Nt01	5126	11	2481	827	91.61	5.13
Nitab4.5_0002229g0180	Nt01	5450	9	1482	494	55.37	4.49
Nitab4.5_0001176g0050	Nt02	4565	10	2103	701	77.47	5.37
Nitab4.5_0001315g0160	Nt04	7912	11	2184	728	81.22	6.11
Nitab4.5_0001180g0060	Nt04	3246	9	2235	745	82.23	7.11
Nitab4.5_0002107g0010	Nt04	5492	9	2274	758	82.43	6.36
Nitab4.5_0004843g0050	Nt06	5529	9	2442	814	88.52	6.41
Nitab4.5_0002442g0010	Nt08	3724	10	2154	718	79.14	6.71
Nitab4.5_0002083g0010	Nt10	6035	10	2007	669	73.61	4.71
Nitab4.5_0000143g0600	Nt11	5690	5	1513	371	70.03	4.74
Nitab4.5_0002888g0060	Nt11	6556	9	2289	763	84.35	5.63
Nitab4.5_0002342g0030	Nt12	6223	8	1692	564	63.32	4.65
Nitab4.5_0000080g0020	Nt13	6576	9	2460	820	90.43	5.11
Nitab4.5_0001198g0350	Nt13	5884	8	1077	359	49.66	4.99
Nitab4.5_0000091g0520	Nt14	4639	10	2154	718	78.73	6.15
Nitab4.5_0002347g0050	Nt17	3697	10	2121	707	77.45	5.83
Nitab4.5_0000351g0080	Nt22	5405	11	2016	672	75.28	6.71
Nitab4.5_0000411g0020	Nt23	5533	11	2484	828	91.77	4.97
Nitab4.5_0002155g0030	Nt23	4841	10	2055	685	75.43	5.99
Nitab4.5_0004973g0030	Nitab4.5_0004973	4848	8	2277	759	83.18	6.08
Nitab4.5_0003509g0020	Nitab4.5_0003509	6627	11	1971	657	71.85	5.31
Nitab4.5_0011619g0010	Nitab4.5_0011619	4670	9	1989	663	72.52	6.27
Nitab4.5_0009124g0030	Nitab4.5_0009124	5337	10	2121	707	77.47	5.78
Nitab4.5_0004851g0070	Nitab4.5_0004851	7277	11	1912	404	73.91	6.66
Nitab4.5_0003066g0010	Nitab4.5_0003066	3284	10	2154	718	79.04	6.72
Nitab4.5_0000631g0120	Nitab4.5_0000631	6547	11	2046	682	74.52	6.17
Nitab4.5_0008369g0020	Nitab4.5_0008369	4116	10	2052	684	74.94	5.34
Nitab4.5_0007217g0030	Nitab4.5_0007217	8378	10	2058	686	76.43	6.57
Nitab4.5_0002049g0010	Nitab4.5_0002049	3562	11	2322	774	66.32	8.03
Nitab4.5_0002214g0130	Nitab4.5_0002214	8470	10	1773	591	66.77	8.35
Nitab4.5_0005109g0040	Nitab4.5_0005109	5319	9	2466	822	89.82	5.98
Nitab4.5_0019165g0010	Nitab4.5_0019165	4817	4	1843	381	52.13	5.31

**Fig. 2:**
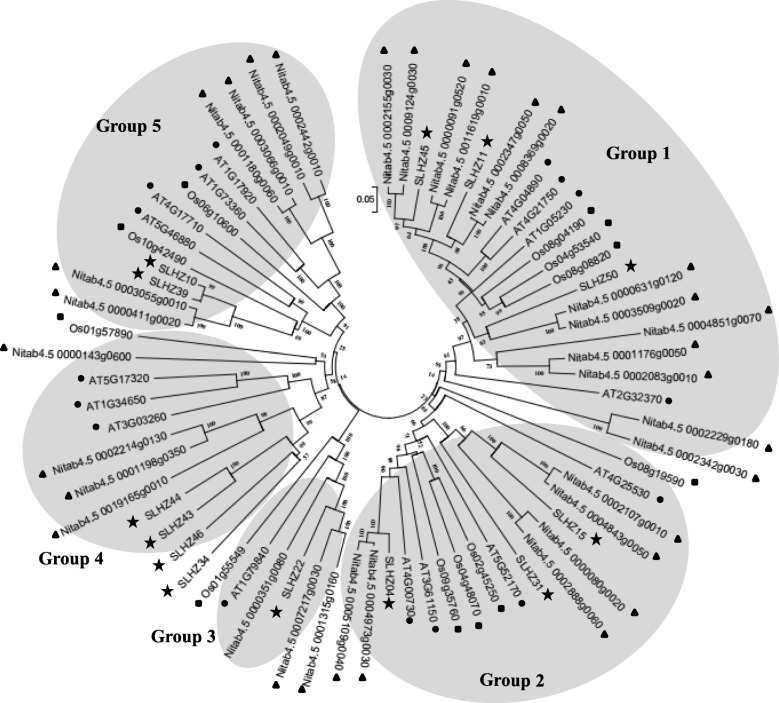
Phylogenetic tree of HD-ZIP IV proteins in different plant species. The protein sequences of 32 *N. tabacum*, 13 tomato, 15 rice, and 16 *Arabidopsis* HD-ZIP IVs were used for the phylogenetic analysis. ▲, *Nicotiana tabacum*; ●, *Arabidopsis thaliana*; ■, *Oryza sativa*; ★, *Solanum lycopersicum*

Evolutionary analysis showed that 74 HD-ZIP IV proteins (32 from tobacco, 13 from tomato, 16 from *Arabidopsis*, and 13 from rice) were clustered into 5 groups (Fig. 2). Each group contained HD-ZIP IVs from the four species. The result of the phylogenetic analysis was consistent with the taxonomic classification: the *HD-ZIP IV* genes from the solanaceous plants (tobacco and tomato) had highly homologous sequences; and the HD-ZIP IVs of eudicots (tobacco, tomato and *Arabidopsis*) were more closely clustered than were those of the monocot (rice).

**Fig. 3:**
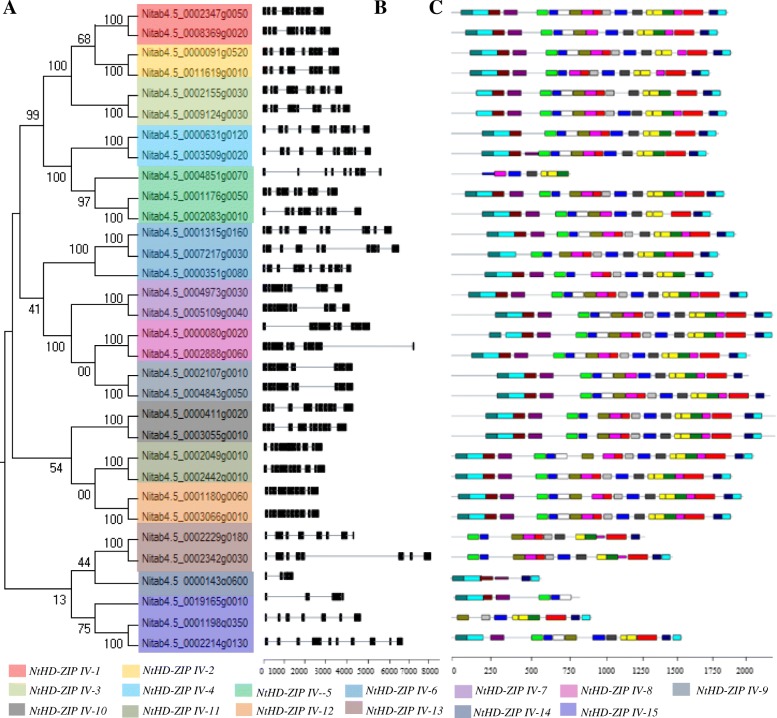
Multiple sequence alignment, gene structure, and conserved motif analysis of *NtHD-ZIP IV*s. **a** Multiple sequence alignment of NtHD-ZIP Vs in *N. tabacum*. **b** Exon-intron structure analysis of *NtHD-ZIP IV* genes. Introns and exons are indicated by black lines and rectangles, respectively. **c** Analysis of the conserved motifs. Conserved motifs are labeled with different colored frames

Gene structure analysis can give insights into the origin and evolution of the HD-ZIP IV gene family in tobacco. A phylogenetic tree was constructed to verify the consistency of the exon-intron pattern and the phylogenetic classification. The tobacco *HD-ZIP IV* genes were divided into 15 categories, which we designate with the prefix “Nt”: *NtHD-ZIP-IV-1* to *NtHD-ZIP-IV-15* (Fig. 3a). The closely related *NtHD-ZIP IV* genes had a similar gene structure. Similarly to the situation found in other plants, the features of the NtHD-ZIP IV gene family varied substantially, and the exon number varied from 4 to 11 (Fig. 3b). It is noteworthy that non intron sequence was inserted in the conserved domain. Analysis of conserved motifs found that 20 motifs were present in the 15 NtHD-ZIP IV proteins (Fig. 3c, Additional file 1: Figure S1). There were usually similar motif patterns in closely-related proteins in the phylogenetic tree, thus indicating evolutionary and functional conservation within a clade.

### Spatial gene expression of *HD-ZIP IVs*

**Fig. 4:**
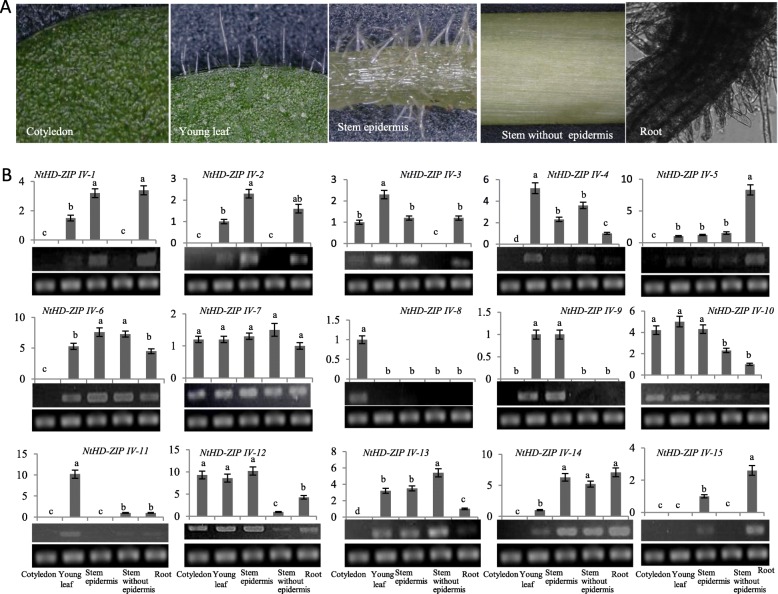
Spatial expressional analysis of *NtHD-ZIP IVs.* **a** Morphological features of the epidermis on various tissues. Scale bar = 100 μm. **b** Gene transcript levels in various tobacco tissues. The lowest transcription for each gene was regarded as a standard, and *L25* gene was taken as endogenous control. Gels: upper, *NtHD-ZIP IV* gene segments amplified by semi-quantitive RT-PCR; lower, *L25* gene segments amplified by semi-quantitive RT-PCR. Data was analyzed using one-way ANOVA followed by least significant difference (LSD) to determine the significance of differences between means using SPSS version 11.0. Each bar represents the average of three biological replicates. Different letters in the same gene indicate significant differences (*P* < 0.05)

The expression pattern of 15 *NtHD-ZIP IVs* in five tobacco tissues was investigated to assess their role in the epidermal development. There were no trichomes on the cotyledons, but many glandular and non-glandular trichomes occurred on the outer surface of leaves and stems (Fig. 4a). As shown in Fig. 4b, *NtHD-ZIP-IV-1* and *NtHD-ZIP-IV-2* were specifically expressed in the leaf, root, and stem epidermis. No expression of these genes was detected in cotyledons and in stems without epidermis. This indicates that *NtHD-ZIP-IV-1* and *NtHD-ZIP-IV-2* are trichome-specific genes. The expression of *NtHD-ZIP-IV-3, NtHD-ZIP-IV-10,* and *NtHD-ZIP-IV-12* was weak in stems without epidermis; this suggests that these three genes may relate to trichome development. Five NtHD-ZIP IV genes (*NtHD-ZIP-IV-4*, *− 5*, *− 6*, *− 13*, and *− 14)*, had similar expression patterns: not expressed in cotyledons, but expressed in leaves, roots, stem epidermis, and stems without epidermis. *NtHD-ZIP-IV-7* showed a consistent transcript level in five tissues. These genes may have complex roles in tobacco development. Notably, *NtHD-ZIP-IV-8* was expressed only in the cotyledons, *NtHD-ZIP-IV-9* only in the leaf and stem epidermis, *NtHD-ZIP-IV-11* only in the leaf, and *NtHD-ZIP-IV-15* only in the root and stem epidermis. These results indicated that each *NtHD-ZIP IV* gene may be associated with the development of different plant organs.

### MeJA application induced the initiation of long-stalk glandular trichomes

**Fig. 5:**
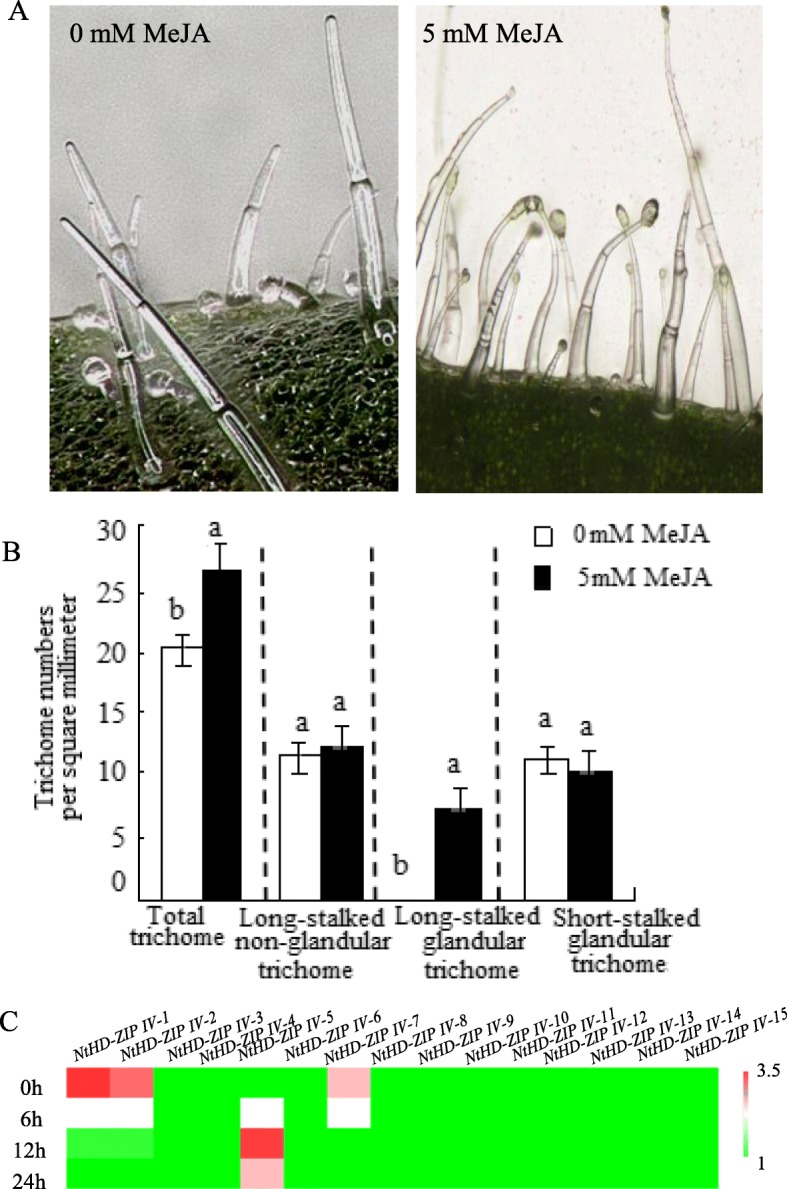
Effects of MeJA on long-stalk glandular trichomes. **a** Exogenous MeJA application induced the initiation of long-stalk glandular trichomes. Scale bar = 100 μm. **b** Trichome density affected by MeJA application. Different letters show significant differences (*P* ≤ 0.05). **c** Transcript levels of *NtHD-ZIP IVs* upon 5.0 mM MeJA application. The lowest transcript level for each gene was regarded as a standard. *L25* was selected as a control gene. The results were calculated by the 2^-ΔΔCT^ method

Only non-glandular and short-stalked glandular trichomes are present on the surface of tobacco T.I.1112 plants. After MeJA application, long-stalk glandular trichomes were observed, and the density was significantly increased with the increase of the MeJA concentration; this was not the case for the non-glandular and short-stalked glandular trichomes (Fig. 5a, b). These results indicated that the morphogenesis of different trichome types was regulated by different networks.

Detecting the transcript level of 15 *NtHD-ZIP-IVs* in the epidermis found that most *NtHD-ZIP IV* genes were not response to MeJA treatment except *NtHD-ZIP-IV-1*, *− 2*, *− 5,* and *− 7. NtHD-ZIP-IV-1*, *− 2*, and *− 7* were inhibited under MeJA application, whereas the transcription level of *NtHD-ZIP-IV-5* increased after MeJA treatment (Fig. 5c).

### Expression pattern of *NtHD-ZIP IV* genes under abiotic stress and hormone treatments

**Fig. 6:**
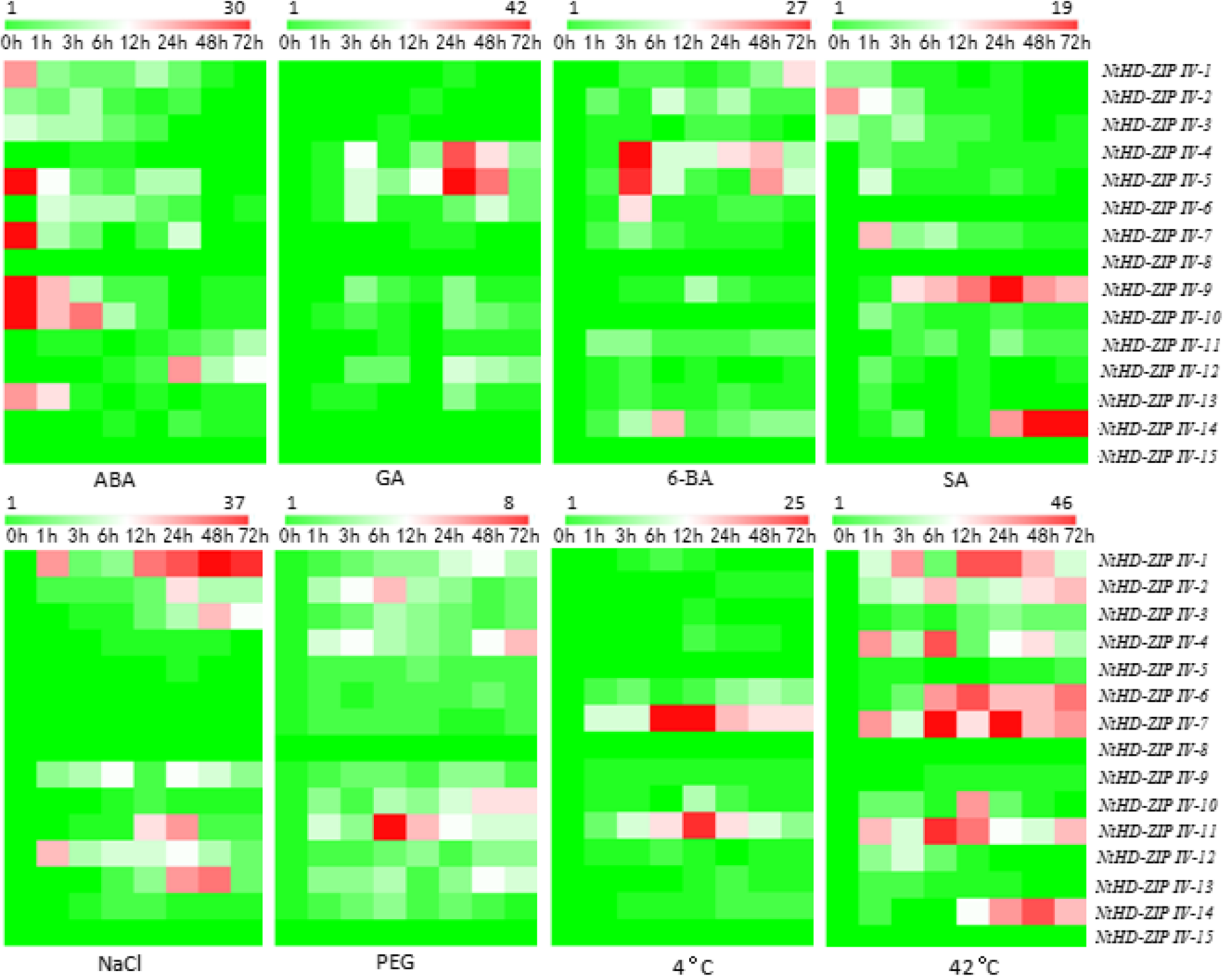
Heat maps of *NtHD-ZIP IVs* gene expression under different hormones and abiotic stresses. qRT-PCR was performed to analyze the transcript level of *NtHD-ZIP IVs*, and the results were calculated by the 2^-ΔΔCT^ method. *L25* was selected as a control gene. The lowest transcript level at each treatment for each gene was set as 1

Plant hormones are key regulators in plant growth and development, as are various environmental stimuli. The *NtHD-ZIP IV* genes had diverse responses to the various hormone treatments (Fig. 6). Following ABA treatment, *NtHD-ZIP-IV-1*, *2*, *− 3*, *− 5*, *− 7*, *− 9*, *− 10*, and *− 13* were inhibited, *NtHD-ZIP-IV-6*, *− 11*, and *− 12* were slightly induced, whereas *NtHD-ZIP-IV-4*, *− 8*, *− 13*, and *− 14* showed no response. GA treatment induced expression of *NtHD-ZIP-IV-4*, *− 5*, *− 6*, *− 9*, *− 10*, *− 12*, and *− 13*, whereas the remaining NtHD-ZIP IV genes showed no response. Similarly, most NtHD-ZIP IV genes were activated by 6-BA treatment (but not *NtHD-ZIP-IV-8*, *− 10*, and *− 15*)*.* Following SA treatment, *NtHD-ZIP-IV-1*, *− 2*, and *− 3* were inhibited, *NtHD-ZIP-IV-6*, *− 8*, and *− 15* did not respond, and the transcript level of the remaining NtHD-ZIP IV genes increased. Compared with other NtHD-ZIP IV genes*, NtHD-ZIP-IV-9* and *-14* could be upregulated at a constant rate by exogenous SA. The findings suggested that *NtHD-ZIP IVs* might be implicated in complex networks, with each member having distinct funtions.

We found that that most *NtHD-ZIP IV*s could be activated by abiotic stress, to varying degrees. *NtHD-ZIP IV* genes were more sensitive to heat stress than to salt, drought, and cold stress. Following high salinity treatment, the expression of *NtHD-ZIP-IV-1*, *− 2*, *− 3*, *− 9*, *− 11*, *− 12*, and *− 13* was upregulated, whereas the remaining genes showed no clear changes. Under drought stress, most of *NtHD-ZIP IV* genes were up-regulated, except for *NtHD-ZIP-IV-8* and -*15.* Conversely, most NtHD-ZIP IV genes were not obviously activated by cold, except for *NtHD-ZIP-IV-6*, *− 7*, *− 10*, and *− 11.* Among the four genes, *NtHD-ZIP-IV-7* and *-11* showed the strongest response to cold stress. Under heat stress, most NtHD-ZIP IV genes were significantly activated, except for *NtHD-ZIP-IV-5*, *− 8*, *− 9*, *− 13*, and *− 15*; in those that were activated, the expression levels were high. Moreover, the transcription levels of *NtHD-ZIP-IV-1*, *− 2*, *− 4*, *− 6*, *− 7*, and *− 11* were high at each sampling occasion after application of heat stress.

## Discussion

### *HD-ZIP IV* genes are conserved during evolution

Plant-specific *HD-ZIP IV*s have been involved in the regulation of epidermal development, including root hairs, trichomes, cuticles and stomates [17]. Sequence analysis indicates that *HD-ZIP IV*s are highly conserved during the evolution or separation processes of various plant species. However, there were different epidermal characteristics in different plant species. Here, we characterized the 15 *NtHD-ZIP IV* genes in the tobacco genome.

There are mainly three principal evolutionary mechanisms of gene duplications: tandem duplication, segmental duplication, and transposition events [41]. In plants, segmental duplication is the most frequent mechanism due to the property of diploidized polyploid [42]. In the present study, some *NtHD-ZIP IV* genes were distributed in duplicated blocks, indicating that segmental duplications have contributed to the gene duplication of *NtHD-ZIP IVs*. The phylogenetic analysis of the HD-ZIP IVs from different species suggested that the HD-ZIP IV duplications within species were first clustered into the same clade, and then grouped together with other species. This finding indicates that *HD-ZIP IV*s diversified and expanded after the radiation of species.

### Some *HD-ZIP IV*s may play crucial roles in the trichome formation

The regulatory network that controls unicellular trichome formation has been well studied in *Arabidopsis* [43, 44]. Similarly to the situation found in the tomato and potato, trichomes in tobacco are typically multicellular structures. Prior to now, there has been a fragmented understanding of the molecular mechanisms underlying multicellular trichome formation. Two paralogous *HD-ZIP IVs* (*HDG11* and *HDG12*) were involved in the trichome branching. Specifically, *hdg11* mutants had more branched trichomes in the leaves, and *hdg12* mutants had more normal trichomes than occurred in the wild type. The excessive-branching morphology of the trichome in *hdg11* mutants was enhanced by *hdg12*, revealing a synergistic effect on the trichome development. In our study, the *HDG11* and *HDG12* homologous genes were *HD-ZIP-IV-11* and *HD-ZIP-IV-12. NtHD-ZIP-IV-11* was expressed only in the leaf. *NtHD-ZIP-IV-12* was strongly expressed in the cotyledons and stem epidermis, whereas weak expression was detected in stems without epidermis. From this, we deduce that *NtHD-ZIP-IV-12* may be related to epidermal development. In tomato, an *HD-ZIP IV* gene (*Wo*) is involved in the initiation of multicellular trichomes [45, 46]. Suppression of *Wo* expression by RNA interference decreases the density of type I trichomes. The homologous gene of *Wo* in *Arabidopsis*, *PDF2*, may regulate shoot epidermal cell differentiation [47]. These results indicate that the formation of multicellular trichomes might be regulated by a distinct network unlike the unicellular trichomes. Further, these *HD-ZIP IV*s may act different roles in the initiation of the unicellular and multicellular trichomes. Here, the predicted proteins coded by the *Wo* gene showed 73, 75, 78, and 79% amino acid sequence identity to the four *Wo* homologs in tobacco, which were further clustered as *NtHD-ZIP-IV-1* and *NtHD-ZIP-IV-2* (Additional file 1: Figure S2). Tissue-preferential expression pattern is an indication of the specific gene function. We found that *NtHD-ZIP-IV-1* and *NtHD-ZIP-IV-2* were trichome-specific genes. Moreover, *NtHD-ZIP-IV-1* and *NtHD-ZIP-IV-2* were strongly upregulated under MeJA application, followed by the initiation of secreting trichomes. Our results indicate that *NtHD-ZIP-IV-1* and *-2* may act crucial roles in the induction of the secreting trichome, similar to the role of *Wo* in tomato.

### Diverse *HD-ZIP IV*s were implicated in hormone and abiotic stress response

In the present study, JA could induce the generation of glandular trichomes. Recently, *HDG11* in *Arabidopsis*, a homologous gene of *NtHD-ZIP-IV-11,* has been reported to control the JA biosynthesis [48]. However, *NtHD-ZIP-IV-11* was not responsive to JA in our present study. The transcripts of most *NtHD-ZIP-IV*s were not respond to MeJA treatment, except for *NtHD-ZIP-IV-1*, *− 2*, *− 5*, and *− 7*, which may play important roles in the induction of secreting trichomes. Surprisingly, most *NtHD-ZIP IVs* responded to ABA, GA, 6-BA, and SA. These hormones are key signaling regulators in plant responses to abiotic stresses [49].

This study primarily focused on determining the dynamic transcriptional changes in *NtHD-ZIP IV* genes under various abiotic stresses. The results indicate that most *NtHD-ZIP IVs* were sensitive to heat, but insensitive to cold and osmotic stress; each *NtHD-ZIP IV* gene had distinct functions; and *NtHD-ZIP IVs* were implicated in a complex network of responses to abiotic stress. The *NtHD-ZIP IV* genes might be good target genes for improving abiotic-stress tolerance in crop plants.

## Conclusions

Fifteen *HD-ZIP IV* genes were identified from *N. tabacum* genome. These *NtHD-ZIP-IVs* showed differential tissue-specific expression patterns. Jasmonates could induce the generation of glandular trichome, and four *NtHD-ZIP-IVs* were implicated in glandular trichome induction. Each *NtHD-ZIP IV* gene had a distinct role in abiotic stress and phytohormone response. The present study provides evidence to elucidate the gene functions of *NtHD-ZIP IVs* in epidermal development and stress responses.

## Methods

### Analysis of the HD-ZIP IV gene family in *N. tabacum*

The sequence of the *Solanum lycopersicum* and *Oryza sativa* HD-ZIP IV gene family was obtained at the Solanaceae Genome Network (https://solgenomics.net/) and the Rice Genome Database (http://rice.plantbiology.msu.edu/), respectively. The *A. thaliana* HD-ZIP IV proteins were obtained using the *Arabidopsis* Information Resource (http://www.arabidopsis.org/). The *Arabidopsis* HD-ZIP IV proteins were used as query seeds to identify the *N. tabacum* HD-ZIP IV proteins (https://solgenomics.net/), via a BlastP search (e < 1^− 10^). These predicted HD-ZIP IV proteins were further confirmed and analyzed using the Pfam tool and SMART web server. The biophysical properties of the HD-ZIP IVs were estimated with the ExPASy ProtParam tool.

To estimate the phylogeny of the *HD-ZIP IV* genes, phylogenetic analysis was carried out using MEGA 7.0 with 1000 replicates, using the HD-ZIP IVs in tobacco, tomato, rice and *Arabidopsis*. Sequences were aligned with ClustalW program. Gene structure was visualized with the Gene Structure Display Server 2.0. The Multiple Expectation Maximization for Motif Elicitation tool was performed to identify the conserved motif. To determine synteny, the synteny blocks containing *HD-ZIP IVs* in the *N. tabacum* genome were scaned using the MCScanX project. The position of each gene in the corresponding chromosome and its synteny relationship were generated using Circos (http://circos.ca/).

### Tissue-specific expression analysis

*N. tabacum* ‘K326’ seedlings were raised in a growth chamber at 22 °C with a 12/12 h light-dark photoperiod. For the tissue-specific expression analysis, cotyledons were sampled from one-week-old seedlings, and the leaf, root, stem epidermis, and the stem with its epidermis removed were sampled from three-week-old seedlings.

Total RNA was extracted and removed the residual DNA using DNase I. Quantitative real-time PCR (qRT-PCR) and semi-quantitative RT-PCR were employed to determine the relative mRNA transcriptions of *HD-ZIP IV*s in five tobacco tissues using the gene-specific primers (Additional file 1: Table S1). *L25* gene was selected as an internal control. q-PCR reaction was performed on an ABI PRISM 7000 system (Applied Biosystems, USA) with the SYBR Green RT-PCR Kit (Takara, China). Each reaction was run in triplicate, and analysis was performed using the 2^-ΔΔCT^ method [50].

### Induction of long-stalk glandular trichomes by MeJA

*N. tabacum* T.I.1112 without long-stalked glandular trichomes was developed by the Oxford Tobacco Research Station. Seedlings at the four-leaf stage were sprayed with 5.0 mM methyl jasmonate (MeJA). Plants were sprayed until all plants were saturated. Three applications were repeated every one week. Three weeks later, three plants from each treatment were selected, and the youngest terminal leaflet at least 5 cm in length on each plant was sampled for the trichome morphology observation. The area of glandular head, and trichome density on the upper leaf surface were counted using an Axioplan 2 microscope (Zeiss, Oberkochen, Germany). The morphological data were analyzed using one-way ANOVA. Moreover, the leaf epidermis of plants exposed to the 5.0 mM MeJA treatment, and of the control, was removed to analyze the expression level of *HD-ZIP IVs*.

### Abiotic stress and hormone treatments

To test the effects of abiotic stress, K326 tobacco seedlings at the four-leaf stage were stressed by placing the plants under one of four treatments: application of 300 mM NaCl or PEG-6000 (− 0.5 MPa) solutions; and exposure to low (4 °C) or high (42 °C) temperatures. In preliminary studies, we found that these treatments caused significant stress to the plants. Control plants were cultured normally without treatment.

To test the effects of exogenous hormone treatment, seedlings at the four-leaf stage were sprayed separately with 100 μM abscisic acid (ABA), 100 μM 6-benzylaminopurine (6-BA), 2.0 mM salicylic acid (SA), and 150 μM gibberellic acid (GA). Control seedlings were sprayed with distilled water. True leaves were collected at 0, 1, 3, 6, 12, 24, 48, and 72 h post treatment for q-PCR analysis.

## Supplementary information

**Additional file 1: Table S1.** Specific primers of *HD-ZIP IV* in qRT-PCR. **Figure S1.** Motif analysis of the NtHD-ZIP IV proteins. The 20 motifs were analyzed using the MEME online tool. Different letters represent the abbreviation of various amino acids. The higher the letter height, the stronger the conservatism of the amino acid at that position. **Figure S2**. Sequence alignment of NtHD-ZIP IV proteins and Wo from *S. lycopersicum*. Alignments were performed using Megalign program of DNAStar. Identical amino acid residues are shared in black background. Dashed lines represent gaps that were introduced to maximize alignment. (DOCX 568 kb)

## Data Availability

All data generated in this study is available as Additional files.

## Abbreviations

6-BA: 6-benzylaminopurine
ABA: Abscisic acid
GA: Gibberellin
HD-ZIP IV: homeodomain-leucine zipper class IV family
MeJA: Methyl jasmonate
ORF: Open reading frame
q-PCR: Quantitative real-time PCR
RT-PCR: Reverse transcription-polymerase chain reaction
SA: Salicylic acid

## References

[1] Lorenzo O, Solano R (2005). Molecular players regulating the jasmonate signaling network. Curr Opin Plant Biol.

[2] Mauch-Mani B, Mauch F (2005). The role of abscisic acid in plant-pathogen interactions. Curr Opin Plant Biol.

[3] Qu Y, Wang YY, Yin QS, Huang LL, Jiang YG, Li GZ, Hao L (2018). Multiple biological processes involved in the regulation of salicylic acid in Arabidopsis response to NO_2_ exposure. Environ Exp Bot.

[4] Gang DR, Wang J, Dudareva N, Nam KH, Simon JE, Lewinsohn E, Pichersky E (2001). An investigation of the storage and biosynthesis of phenylpropenes in sweet basil. Plant Physiol.

[5] Kroumova AB, Wagner GJ (2003). Different elongation pathways in the biosynthesis of acyl groups of trichome exudates sugar esters from various solanaceous plants. Planta.

[6] Zhang H, Zhang S, Yang Y, Jia H, Cui H (2018). Metabolic flux engineering of cembratrien-ol production in both the glandular trichome and leaf mesophyll in *Nicotiana tabacum*. Plant Cell Physiol.

[7] Schilmiller AL, Last RL, Pichersky E (2008). Harnessing plant trichome biochemistry for the production of useful compounds. Plant J.

[8] Rios-Estepa R, Turner GW, Lee JM, Croteau RB, Lange BM (2008). A systems biology approach identifies the biochemical mechanisms regulating monoterpenoid essential oil composition in peppermint. Proc Natl Acad Sci U S A.

[9] Traw MB, Bergelson J (2003). Interactive effects of jasmonic acid, salicylic acid, and gibberellin on induction of trichomes in Arabidopsis. Plant Physiol.

[10] Boughton AJ, Hoover K, Felton GW (2005). Methyl jasmonate application induces increased densities of glandular trichomes on tomato, *Lycopersicon esculentum*. J Chem Ecol.

[11] Perotti MF, Ribone PA, Chan RL (2017). Plant transcription factors from the homeodomain-leucine zipper family I. role in development and stress responses. IUBMB Life.

[12] Roodbarkelari F, Groot EP (2016). Regulatory function of homeodomain-leucine zipper (HD-ZIP) family proteins during embryogenesis. New Phytol.

[13] Yan T, Li L, Xie L, Chen M, Shen Q, Pan Q, Fu X, Shi P, Tang Y, Huang H (2018). A novel HD-ZIP IV/MIXTA complex promotes glandular trichome initiation and cuticle development in Artemisia annua. New Phytol.

[14] Ruberti I, Sessa G, Lucchetti S, Morelli G (1991). A novel class of plant proteins containing a homeodomain with a closely linked leucine zipper motif. EMBO J.

[15] Johannesson H, Wang Y, Engström P (2001). DNA-binding and dimerization preferences of Arabidopsis homeodomain-leucine zipper transcription factors in vitro. Plant Mol Biol.

[16] Sessa G, Carabelli M, Ruberti I, Lucchetti S, Baima S, Morelli G (1994). Identification of distinct families of HD-ZIP proteins in *Arabidopsis thaliana*. Plant Mol Biol.

[17] Ariel FD, Manavella PA, Dezar CA, Chan RL (2007). The true story of the HD-zip family. Trends Plant Sci.

[18] Javelle M, Vernoud V, Rogowsky PM, Ingram GC (2011). Epidermis: the formation and functions of a fundamental plant tissue. New Phytol.

[19] Nakamura M, Katsumata H, Abe M, Yabe N, Komeda Y, Yamamoto KT, Takahashi T (2006). Characterization of the class IV homeodomain-leucine zipper gene family in Arabidopsis. Plant Physiol.

[20] Zalewski CS, Floyd SK, Furumizu C, Sakakibara K, Stevenson DW, Bowman JL (2013). Evolution of the class IV HD-zip gene family in Streptophytes. Mol Biol Evol.

[21] Yu H, Chen X, Hong YY, Wang Y, Xu P, Ke SD, Liu HY, Zhu JK, Oliver DJ, Xiang CB (2008). Activated expression of an Arabidopsis HD-START protein confers drought tolerance with improved root system and reduced stomatal density. Plant Cell.

[22] Chen E, Zhang X, Yang Z, Wang X, Yang Z, Zhang C, Wu Z, Kong D, Liu Z, Zhao G (2017). Genome-wide analysis of the HD-ZIP IV transcription factor family in Gossypium arboreum and GaHDG11 involved in osmotic tolerance in transgenic Arabidopsis. Mol Gen Genomics.

[23] Wang S, Kwak SH, Zeng Q, Ellis BE, Chen XY, Schiefelbein J, Chen JG (2007). TRICHOMELESS1 regulates trichome patterning by suppressing GLABRA1 in Arabidopsis. Development.

[24] Masucci JD, Rerie WG, Foreman DR, Zhang M, Galway ME, Marks MD, Schiefelbein JW (1996). The homeobox gene GLABRA2 is required for position-dependent cell differentiation in the root epidermis of *Arabidopsis thaliana*. Development.

[25] Kamata N, Okada H, Komeda Y, Takahashi T (2013). Mutations in epidermis-specific HD-ZIP IV genes affect floral organ identity in *Arabidopsis thaliana*. Plant J.

[26] Ogawa E, Yamada Y, Sezaki N, Kosaka S, Kondo H, Kamata N, Abe M, Komeda Y, Takahashi T (2015). ATML1 and PDF2 play a redundant and essential role in Arabidopsis embryo development. Plant Cell Physiol.

[27] Kubo H, Peeters AJ, Aarts MG, Pereira A, Koornneef M (1999). ANTHOCYANINLESS2, a homeobox gene affecting anthocyanin distribution and root development in Arabidopsis. Plant Cell.

[28] Fu R, Liu W, Li Q, Li J, Wang L, Ren Z (2013). Comprehensive analysis of the homeodomain-leucine zipper IV transcription factor family in Cucumis sativus. Genome.

[29] Belamkar V, Weeks NT, Bharti AK, Farmer AD, Graham MA, Cannon SB (2014). Comprehensive characterization and RNA-Seq profiling of the HD-zip transcription factor family in soybean (*Glycine max*) during dehydration and salt stress. BMC Genomics.

[30] Javelle M, KleinCosson C, Vernoud V, Boltz V, Maher C, Timmermans M, DepègeFargeix N, Rogowsky PM (2011). Genome-wide characterization of the HD-ZIP IV transcription factor family in maize: preferential expression in the epidermis. Plant Physiol.

[31] Gao Y, Gao S, Xiong C, Yu G, Chang J, Ye Z, Yang C (2015). Comprehensive analysis and expression profile of the homeodomain leucine zipper IV transcription factor family in tomato. Plant Physiol Bioch.

[32] Wagner GJ (1991). Secreting glandular trichomes: more than just hairs. Plant Physiol.

[33] Kennedy BS, Nielsen MT, Severson RF (1995). Biorationals from nicotiana protect cucumbers against *Colletotrichum-Lagenarium* (pass) ell & halst disease development. J Chem Ecol.

[34] Lin Y, Wagner GJ (1994). Surface disposition and stability of pest-interactive, trichome-exuded diterpenes and sucrose esters of tobacco. J Chem Ecol.

[35] Liu X, Zhang J, Liu Q, Tang G, Wang H, Fan C, Yin S. Bioactive cembranoids from the South China Sea soft coral *Sarcophyton elegans*. Molecules. 2015:13324–35. 10.3390/molecules200713324 PMC6331945 26205057

[36] El Sayed KA, Sylvester PW (2007). Biocatalytic and semisynthetic studies of the anticancer tobacco cembranoids. Expert Opin Investig Drugs.

[37] Duan S, Du Y, Hou X, Li D, Ren X, Dong W, Zhao W, Zhang Z (2015). Inhibitory effects of tobacco extracts on eleven phytopathogenic fungi. Nat Prod Res Dev.

[38] Zubair MS, Anam S, Al-Footy KO, Abdel-Lateef A, Alarif WM (2014). Cembranoid diterpenes as antitumour: molecular docking study to several protein receptor targets. Proc Int Conf Comput Sci Technol.

[39] Martins AH, Hu J, Xu Z, Mu C, Alvarez P, Ford BD, El Sayed K, Eterovic VA, Ferchmin PA, Hao J (2015). Neuroprotective activity of (1S,2E,4R,6R,-7E,11E)-2,7,11-cembratriene-4,6-diol (4R) in vitro and in vivo in rodent models of brain ischemia. Neuroscience.

[40] Ning Y, Du Y, Liu X, Zhang H, Liu Y, Shi J, Xue SJ, Zhang Z (2017). Analyses of effects of α-cembratrien-diol on cell morphology and transcriptome of Valsa Mali var. Mali. Food Chem.

[41] Gu Z, Steinmetz LM, Gu X, Scharfe C, Davis RW, Li W (2003). Role of duplicate genes in genetic robustness against null mutations. Nature.

[42] Cannon SB, Mitra A, Baumgarten A, Young ND, May G (2004). The roles of segmental and tandem gene duplication in the evolution of large gene families in *Arabidopsis thaliana*. BMC Plant Biol.

[43] Szymanski DB, Lloyd AM, Marks MD (2000). Rogress in the molecular genetic analysis of trichome initiation and morphogenesis in Arabidopsis. Trends Plant Sci.

[44] Larkin JC, Brown ML, Schiefelbein J (2003). How do cells know what they want to be when they grow up? Lessons from epidermal patterning in Arabidopsis. Annu Rev Plant Biol.

[45] Yang C, Li H, Zhang J, Luo Z, Gong P, Zhang C, Li J, Wang T, Zhang Y, Lu YE (2011). A regulatory gene induces trichome formation and embryo lethality in tomato. Proc Natl Acad Sci U S A.

[46] Gao S, Gao Y, Xiong C, Yu G, Chang J, Yang Q, Yang C, Ye Z. The tomato B-type cyclin gene, SlCycB2, plays key roles in reproductive organ development, trichome initiation, terpenoids biosynthesis and Prodenia litura defense. Plant Sci. 2017:103–14. 10.1016/j.plantsci.2017.05.006 28716406

[47] Abe M, Katsumata H, Komeda Y, Takahashi T (2003). Regulation of shoot epidermal cell differentiation by a pair of homeodomain proteins in Arabidopsis. Development.

[48] Cai XT, Xu P, Wang Y, Xiang CB (2015). Activated expression of AtEDT1/HDG11 promotes lateral root formation in Arabidopsis mutant edt1 by upregulating jasmonate biosynthesis. J Integr Plant Biol.

[49] Gómez-Cadenas A, Ollas CD, Manzi M, Arbona V, Tran LS, Pal S (2014). Phytohormonal crosstalk under abiotic stress. Phytohormones: a window to metabolism, signaling and biotechnological applications.

[50] Livak KJ, Schmittgen TD (2001). Analysis of relative gene expression data using real-time quantitative PCR and the 2(−Delta Delta C(T)) method. Methods.

