# Supplementary material for: Genome-wide characterization of NtHD-ZIP IV: different roles in abiotic stress response and glandular Trichome induction
Source: BMC Plant Biol. 2019 Oct 24;19:444. doi: 10.1186/s12870-019-2023-4 (PMC6814048; doi:10.1186/s12870-019-2023-4)
Supplement: Supplementary file 1 — Additional file 1: Table S1. Specific primers of HD-ZIP IV in qRT-PCR. Figure S1. Motif analysis of the NtHD-ZIP IV proteins. The 20 motifs were analyzed using the MEME online tool. Different letters represent the abbreviation of various amino acids. The higher the letter height, the stronger the conservatism of the amino acid at that position. Figure S2. Sequence alignment of NtHD-ZIP IV proteins and Wo from S. lycopersicum. Alignments were performed using Megalign program of DNAStar. Identical amino acid residues are shared in black background. Dashed lines represent gaps that were introduced to maximize alignment. (DOCX 568 kb) [file 12870_2019_2023_MOESM1_ESM.docx]

**Genome-Wide Characterization of NtHD-ZIP IV: Different Roles in Abiotic Stress Response and Glandular Trichome Induction**

Hongying Zhang ^1,†^, Xudong Ma^1,†^, Wenjiao Li ^1^, Dexin Niu ^1^, Zhaojun Wang ^1^, Xiaoxiao Yan ^1^, Xinling Yang ^2^, Yongfeng Yang^2^ and Hong Cui ^1, *^

^1^ College of Tobacco Science, Henan Agricultural University, Zhengzhou 450002,
China
^2^ Technology Center, China Tobacco Henan Industrial Co., Ltd., Zhengzhou 450000,
China

^†^ The authors contributed equally to this work

^*^Corresponding author: Hong Cui

Email: [cuihonger_13@163.com](mailto:cuihonger_13@163.com)

Tel/Fax: 86-10-63555763

**Table S1.** Specific primers of *HD-ZIP IV* in qRT-PCR

| Primer name | Forward primer (5´→ 3´) | Reverse primer (5´→ 3´) |
| --- | --- | --- |
| *NtHD-ZIP IV-1* | AAGCGTTACCACCGCCATAC | TTGAGCCTTCATTTGAGTGCG |
| *NtHD-ZIP IV-2* | AATGGAATCGTTTTTCAAAGAGTG | AGTTTGGACATGTAGCATTGCC |
| *NtHD-ZIP IV-3* | ATGGCTCAATCTGGAGAACCC | TTTCCTGCTACGCCAGTTGAC |
| *NtHD-ZIP IV-4* | GTTATCACCGCCATACTCAGCA | GACATTTCGCCAATAGGGGG |
| *NtHD-ZIP IV-5* | ATTTCAAGTACCATTTCCACAGG | AATCCAACGCTTTGCCCC |
| *NtHD-ZIP IV-6* | GGGAAAATACCCAATAGGGACAT | GCTTCTGATCCACAGTGGTTCTC |
| *NtHD-ZIP IV-7* | TCTTCAAGGGATCACACGGC | CGCTCTTTCGGGTCATAACAC |
| *NtHD-ZIP IV-8* | CAATCGTTGTCGTAATCAGGCT | GCAGGATTTGCTATAACGGAAG |
| *NtHD-ZIP IV-9* | TGGTGGCTGATAGTCCATACAAC | TGGTATTTCTTTTTTCTGGATGATT |
| *NtHD-ZIP IV-10* | TTCCCCCATTCAGAACCCTAA | CAGTGTGCCGATGATAACGCT |
| *NtHD-ZIP IV-11*  *NtHD-ZIP IV-12*  *NtHD-ZIP IV-13*  *NtHD-ZIP IV-14*  *NtHD-ZIP IV-15* | CGTTTCCATCGGCACACAG  TGGTGTGATGGGAAGCCG GCCCAGTTCACTCCGTCGT  GACGGTTCAAAATCATCACGA GTGGGTGAGTTAGCCCGAAT | GGCTTCCCTTATTGCTATGTTTT  TATTGCCCACGAGCTTTGC  AGTTTGAGTCTGTTGGCGGAA GTTGCCTCACCTCAAACTCTT  GCAAGGCGACCATCAGGAC |


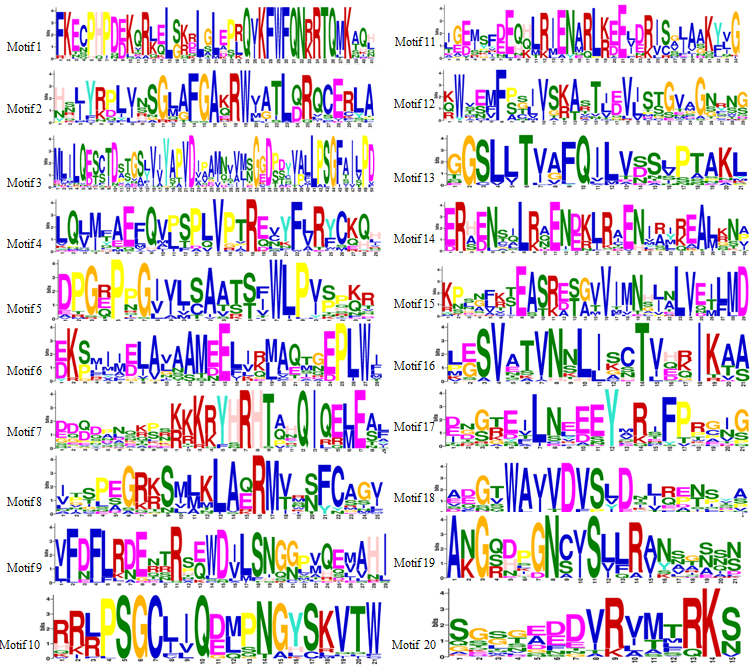


**Figure S1. Motif analysis of the NtHD-ZIP IV proteins.** The 20 motifs were analyzed using the MEME online tool. Different letters represent the abbreviation of various amino acids. The higher the letter height, the stronger the conservatism of the amino acid at that position.


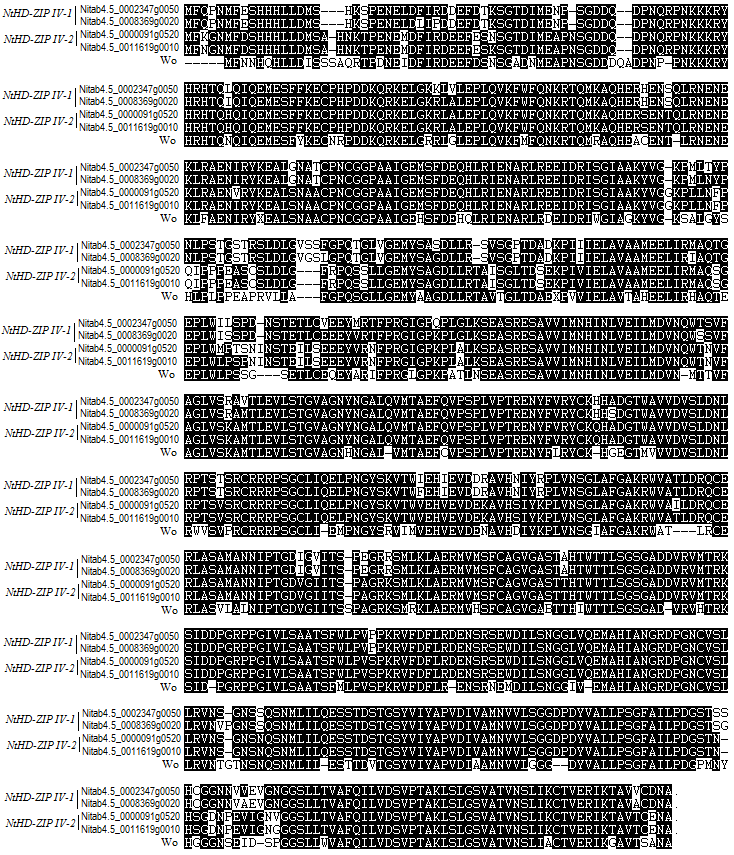


**Figure S2. Sequence alignment of NtHD-ZIP IV proteins and Wo from *S. lycopersicum*.** Alignments were performed using Megalign program of DNAStar. Identical amino acid residues are shared in black background. Dashed lines represent gaps that were introduced to maximize alignment.
